# Classifying Food Items During an Eating Occasion: A Machine Learning Approach with Slope Dynamics for Windowed Kinetic Data

**DOI:** 10.3390/foods14020276

**Published:** 2025-01-16

**Authors:** Ileana Baldi, Corrado Lanera, Mohammad Junayed Bhuyan, Paola Berchialla, Luca Vedovelli, Dario Gregori

**Affiliations:** 1 Unit of Biostatistics, Epidemiology and Public Health, Department of Cardiac, Thoracic, Vascular Sciences and Public Health, University of Padova, 35131 Padova, Italy; ileana.baldi@ubep.unipd.it (I.B.); corrado.lanera@ubep.unipd.it (C.L.); mohammad.bhuyan@ubep.unipd.it (M.J.B.); luca.vedovelli@ubep.unipd.it (L.V.); 2 Department of Clinical and Biological Sciences, University of Torino, 10043 Orbassano, Italy; paola.berchialla@unito.it

**Keywords:** wearable devices, dietary monitoring, acceleration data, kinetic data, random forest, food recognition

## Abstract

Background: Wearable devices equipped with a range of sensors have emerged as promising tools for monitoring and improving individuals’ health and lifestyle. Objectives: Contribute to the investigation and development of effective and reliable methods for dietary monitoring based on raw kinetic data generated by wearable devices. Methods: This study uses resources from the NOTION study. A total of 20 healthy subjects (9 women and 11 men, aged 20–31 years) were equipped with two commercial smartwatches during four eating occasions under semi-naturalistic conditions. All meals were video-recorded, and acceleration data were extracted and analyzed. Food recognition on these features was performed using random forest (RF) models with 5-fold cross-validation. The performance of the classifiers was expressed in out-of-bag sensitivity and specificity. Results: Acceleration along the x-axis and power show the highest and lowest rates of median variable importance, respectively. Increasing the window size from 1 to 5 s leads to a gain in performance for almost all food items. The RF classifier reaches the highest performance in identifying meatballs (89.4% sensitivity and 81.6% specificity) and the lowest in identifying sandwiches (74.6% sensitivity and 72.5% specificity). Conclusions: Monitoring food items using simple wristband-mounted wearable devices is feasible and accurate for some foods while unsatisfactory for others. Machine learning tools are necessary to deal with the complexity of signals gathered by the devices, and research is ongoing to improve accuracy further and work on large-scale and real-time implementation and testing.

## 1. Introduction

Noncommunicable diseases (NCDs) are responsible for almost 41 million deaths (74% of all deaths globally) each year from cardiovascular events, respiratory pathologies, cancer, and diabetes. In high- and middle-income countries, NCDs are mostly related to unhealthy diets, sedentary lifestyles, tobacco smoke, and alcohol abuse. The low consumption of healthy foods, such as fruits and vegetables, replaced by foods rich in fats and sugar, the so-called “junk” foods, is the primary concern of current health policies [1]. To prevent the worsening of chronic diseases and reduce associated economic costs, the development of intelligent and live monitoring systems for caloric intake is becoming necessary as the number of pathologies related to eating increases [2].

Automatic monitoring of food intake has become an important area of research, aiming to tackle the well-known issues of traditional self-reported dietary assessments. Technologies such as image-based caloric analysis, audio-based ingestion recognition, and utensil-based detection have become the most popular methods for tracking food intake. For example, image-based systems use machine learning algorithms to detect food components and estimate portion sizes based on images or videos of meals [3]. Although these techniques can be quite accurate, they depend on users to regularly take sharp images of their food, which can be inconvenient and affected by factors like poor lighting or camera quality. Similarly, audio-based monitoring detects eating events by analyzing the sounds of chewing and swallowing, often using microphones placed near the mouth [4]. Although this approach does not require manual effort during meals, it can feel intrusive and raise privacy concerns in social situations. Utensil-based methods take a different approach, embedding sensors into everyday tools like forks and spoons to track meal frequency and identify foods being consumed [5].

Despite their innovation and accuracy, these methods face significant challenges. Most require expensive, specialized devices—whether it is high-resolution cameras, sensitive microphones, or smart utensils—which can make them inaccessible for large-scale use. They also tend to be invasive, demanding consistent user participation or lifestyle adjustments that may feel unnatural or burdensome. For example, having to take photos before every meal or wear a microphone near your mouth can disrupt daily routines and discourage long-term use. Additionally, these tools may cause discomfort or stigma in social settings, where using such visible technology might draw unwanted attention. As a result, while these methods work well in controlled studies, they often struggle to fit into real-world scenarios or scale to larger populations [3,4,5]. This underscores the need for new approaches—ones that are affordable, noninvasive, and seamlessly integrate into everyday life without sacrificing accuracy or usability.

Food consumption involves specific hand movements, which vary in quality, speed, direction, and interval between bites depending on the type of food; the hypothesis at the base of this study is that wrist kinetic metrics, measured via smartwatch, may provide helpful information for classifying the food consumed. Moreover, to encourage self-monitoring of eating habits and to make it as natural and less invasive as possible, analysis of wrist movement using inertial sensors integrated into wearable devices can also be carried out to study the caloric intake of subjects [6]. Currently, these kinds of studies use kinetic data, that is, accelerometer or gyroscope, analyzing just the behavior of the hands up and down to detect eating occasions [5,6].

This study aims to contribute to the investigation and the development of more robust, effective, and suitable methods for dietary monitoring at a population level. The need for such tools has been widely recommended within the nutrition community [7]. The purpose of this study is to expand existing activity modeling techniques to classify different food items by capturing raw (5 Hz) kinetic signals from a triaxial accelerometer worn on the wrist. In particular, this study explores the use of data from accelerometers worn on the wrist to classify different types of food consumed based on the assumption that hand movements during the act of eating are correlated with specific food characteristics.

## 2. Materials and Methods

### 2.1. Participants

**Figure 1:**
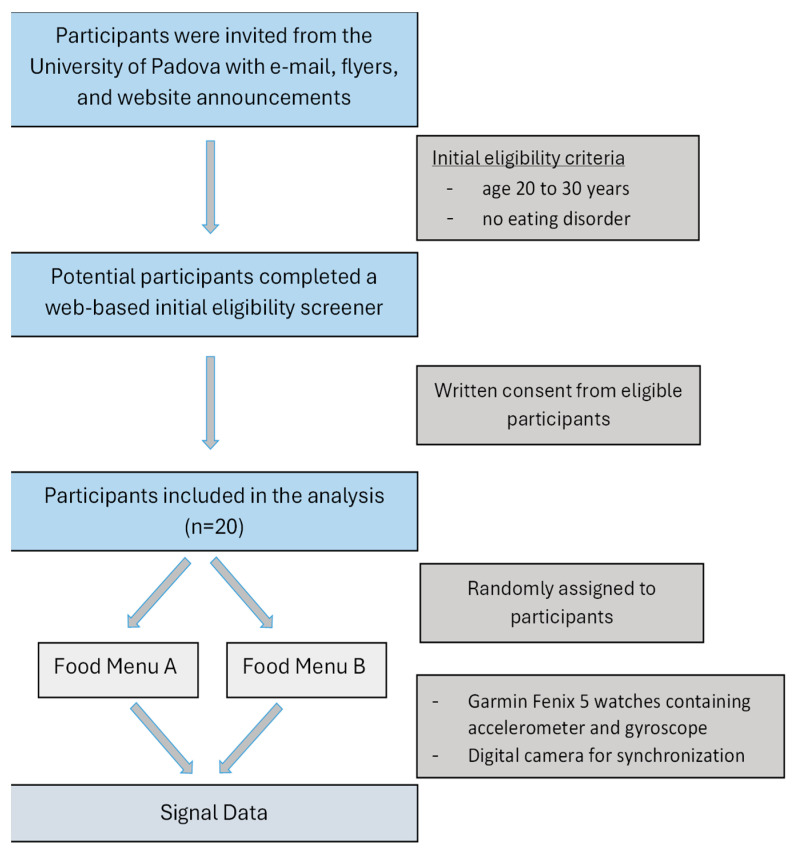
NOTION study design and flow chart of its step.

This study uses data from the NOTION study (acronym of ‘measuriNg calOric inTake at populatION level’) [8]. The Bioethics Committee of the University of Torino (Italy), acting as an Institutional Review Board, approved the study protocol. A total of 20 healthy young subjects (9 women, 11 men) aged 20–30 years completed this study (Table 1). Subjects were free of allergies or other food-related medical conditions (Figure 1).

### 2.2. Experimental Procedures and Data Collection

The NOTION study collected kinetic raw data from two Fenix^®^ 5 wearable Garmin^®^ (Schaffhausen, Switzerland) watches, worn simultaneously on both wrists of each participant under semi-naturalistic conditions. The Garmin Fenix^®^ 5 offers efficient processing, battery life up to approximately 20–22 h with GPS and heart rate monitoring enabled, and a user-friendly interface, enhancing real-world usability. According to the study protocol, participants were randomly assigned to two different daily menus consisting of four meals and different items of food per meal (Table 2).

The Fenix^®^ 5 watches and the camera in use could not electronically synchronize with each other. Therefore, the raw data collection was based on the internal clock of each device in an independent way and aligned in post-production. The devices stored raw data at a rate of 5 Hz by watches and 25 FPS (frame per second) by the camera. The camera recorded both audio and video. Subjects were asked to perform a single hand clap at the beginning of each food-item consumption to allow synchronization between the devices’ clocks. The peaks of the distinct sinusoidal patterns at the beginning of each kinetic raw data subset collected for a food-item meal of a subject of the watches’ raw data and the peak of the corresponding sound channel on the raw data of the camera were aligned in post-production to obtain the start moments for each food-item meal activity.

Two independent evaluators watched the videos to identify all the eating complete-movement applying the following rule: starting and stopping time for an eating complete-movement are the nearest moments, respectively, before and after food items were placed into the mouth. Then, two different sets of labels, ‘eating’/‘non-eating’ labels (‘eating’ was considered as the positive one), were added to each line of raw data according to the opinion of the corresponding evaluator.

We refer, united, to the subset of raw data labeled ‘eating’ by at least one evaluator and intersected the subset of raw data labeled ‘eating’ by both evaluators.

### 2.3. Sensors’ Signals

The raw data provided by Fenix^®^ 5 watches are in FIT (Flexible and Interoperable Data Transfer) file format [9]. The related FIT-SDK software v.20 development kit provides the FitCSVTool.jar JavaTM program, which can convert FIT files in the common CSV format (Comma Separate Value). Converted raw data were merged altogether and imported into R [10], powered by the tidyverse (v.2.0) bundle of packages, and the caret package (v.6.0) [11,12].

From CSV files, we retained kinetic raw data about the x-/y-/z-axis acceleration (reported in milli-G, where G is the Earth’s gravity force), the pitch (reported in radiants, and internally computed by the Fenix^®^ 5 using the equation $atan2\left(y,\;\sqrt{x^{2}+z^{2}}\right)$, where, on an xy-plane, $atan2\left(y,\;x\right)$ the angle between the positive x-axis and the vector (x,y)), the roll (reported in radians, and internally computed by the Fenix^®^ 5 by using the equation $atan2\left(-x,\;z\right)$), and the power (reported in milli-G, it is the Euclidean norm, or magnitude, of the acceleration 3-dimensional vector).

The arm’s linear acceleration and total power estimation are all affected by gravity according to the following equation:

$$power_{total}=\sqrt{gravity^{2}+\sum_{\left\{i=1\right\}}^{3}{\left(axis_{i}\;arm\;linear\;acceleration\right)}^{2}}$$

With the raw data stored/computed/provided by Fenix^®^ 5, it was impossible to precisely extract/compute the nominal linear acceleration for the individual axes. The application of high-pass filters or other more complex techniques could estimate them, but none of them could lead to excellent results [13]. On the other hand, the net total energy expenditure is straightforwardly extracted by the square root of the absolute difference from the square of the power and 1 G^2^. Therefore, we computed this kinetic quantity and added it to the set of variables considered. We did not consider other quantities or manipulations of the raw data. We call *kinetic variables* the final set of these seven pieces of information, i.e., x-/y-/z-axes, pitch, roll, power, and total energy data.

### 2.4. Datasets

**Figure 2:**
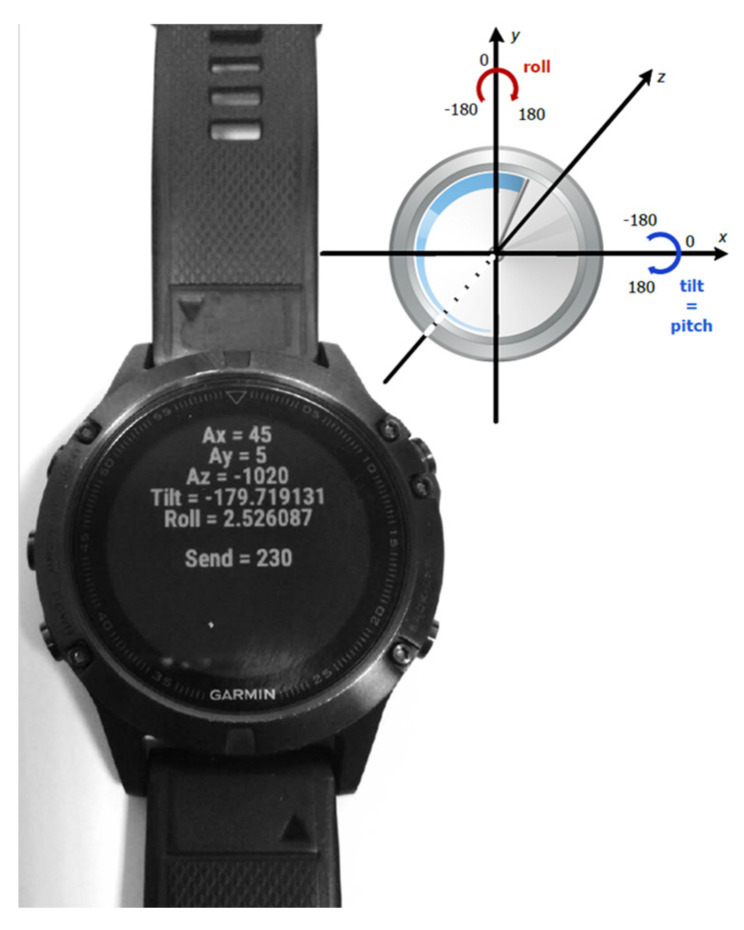
The Garmin^®^ Fenix^®^ 5 reports, among others, the value of the acceleration force on it along three axes, i.e., x, y and z, reported in milli G (where G is the Earth’s gravity force), and the value of roll (rotation around the y-axis) and pitch (rotation around x-axis, also called tilt in the picture), reported in radians. For the NOTION study, the measure of total power (not reported in the picture), i.e., the Euclidean norm, or magnitude, of the acceleration 3-dimensional vector, and the total energy expenditure (the power purged from the gravity force) were also considered for the analyses.

The raw data provided by Fenix^®^ 5 were at 5 Hz rate, i.e., 5 pieces of raw data *per* second (Figure 2). We refer, as *a window, to any consecutive sequence of raw data of a given time-width*. We aggregated the raw data collected by each Fenix^®^ 5 in multiple ways according to the fixed time-width for the window. The widths were 1, 2, 3, 4, and 5 s, i.e., 5, 10, 15, 20, and 25 consecutive pieces of raw data for each window, respectively. For all widths considered, all possible windows of raw data were collected and summarized into a single Information Unit (IU) by considering the slopes of the regression line of the corresponding kinetic variables against time, estimated along the window itself.

The IUs were next labeled as ‘eating’ movement with two possibly different labels. The first label ’eating‘ was assigned to IUs if they contained more than 50% of the pieces of raw data with an ’eating‘ *united* label. The second label ’eating’ was assigned to IU if the same happened for the *intersected* labels. We refer to these two sets of labels for IUs as *united* and *intersected* label criteria.

The analyses were conditioned only on the two possible sets of ’eating’ IU, that is, the *united* IU and the *intersected* IU, and stratified by the time-width of the considered window. We also stratified the analyses using Fenix^®^ 5 considered as a source of raw data. We refer to as full the condition of using both Fenix^®^ 5 as a raw data source and *dominant* the condition of using only one Fenix^®^ 5 worn on the subject’s dominant arm as a raw data source.

Finally, we performed 20 different stratified analyses considering all combinations of time-widths, criteria to label IU as ’eating’, and sources of raw data (Table 3).

### 2.5. Model

We implemented random forest (RF)-based multiclassification algorithms to identify a set of 13 food items on four meals [14]. The RF is an algorithm based on the growth of numerous decision trees that make decisions by a system of votes [15]. The analyst decides the number of trees a priori, and it should be big enough to achieve stability in the votes. Once the stability of the votes is achieved, increasing the number of the trees is useless for improving the model, as it only leads to higher computational intensity [16]. The forest selects some samples from the original dataset based on a bootstrap strategy, i.e., resampling with replacement to fit each tree of the model [17]. Furthermore, each tree is bound to sample only a fixed number of variables for training. This number of variables, often called the mtry parameter, is also provided a priori by the analyst, and can range from 1 to the total number of variables.

### 2.6. Performance Estimation

We used a Cross-Validation (CV) strategy to select the optimal mtry and obtain the best model, which avoids the problem of over-fitting [18]. For each stratified analysis, we performed 5-fold CV searching across the whole set of possible values for the mtry parameter, growing 500 trees in each forest. The criterion considered for the selection of the parameter was the maximization of the global validation accuracy (i.e., the proportion of correctly predicted samples in the validation set). Finally, we selected the optimal mtry and grew the final forest for each set of forests grown during the CV step. The final performances are the one-vs.-all sensitivity (proportion of true positives over the positives) and specificity (proportion of true negatives over the negatives). We computed them on the Out-Of-Bag (OOB) samples, i.e., for each food item, we computed the two mentioned statistics, “asking” each tree of each forest to answer the question “Is this a sample of eating occasions involving this specific food item?”. We repeated it for each sample in each dataset not involved in the training of that tree. In the Supplementary Materials (Tables S1–S4), we report the results on the side of the corresponding window size and the optimal mtry parameter, selected by the CV procedure. We also reported patterns for sensitivity/specificity changes with respect to the changing of the time-width, and is stratified by label criteria, source of raw data, and food item.

### 2.7. Variables’ Importance

**Figure 3:**
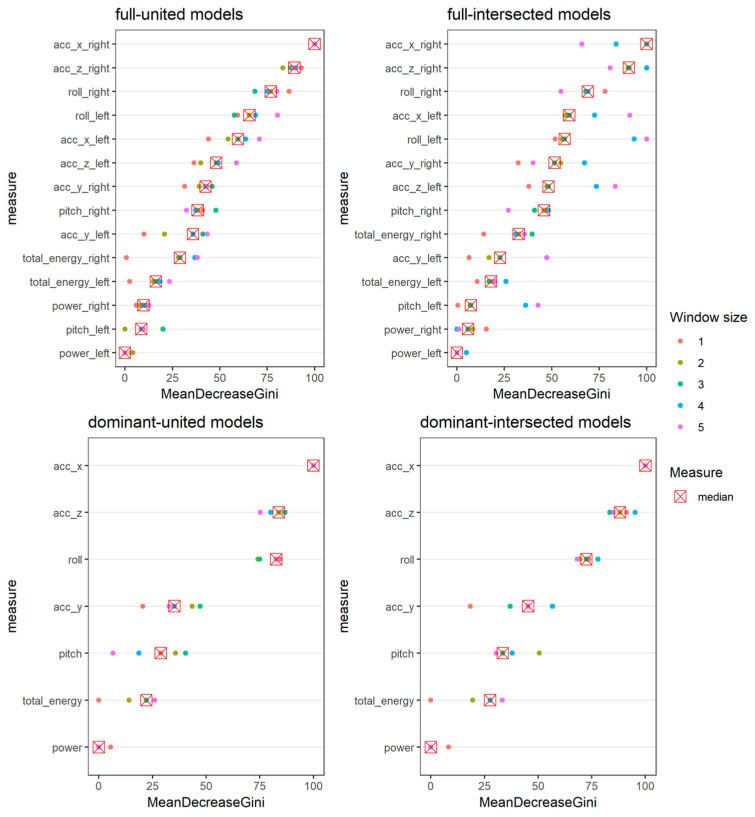
Variable importances of the kinetic variables reported as mean decrease Gini index scaled on 0–100 for all the twenty stratified random forest models trained (dots), and for their corresponding time-width medians (crosses) stratified by the source of raw data (i.e., full or dominant, by panels’ rows) and by labeling criteria (i.e., united or intersected, by panels’ columns).

The importance of the variables in the models was also assessed by averaging over all the trees the decrease in the node Gini impurity index for each variable, which is $\sum_{i=1}^{n}p_{i}(1-p_{i})$, i.e., the sum of each observation (i) of the products to be correctly classified $p_{i}$ and to being wrongly classified $\left(1-p_{i}\right)$ if a split is performed according to the interested variable. Figure 3 reports the results of the variable importance investigation (scaled in the range 0–100).

## 3. Results

Watches generated 278,260 pieces of raw data, each consisting of seven directly provided or computed kinetic variables, i.e., the x-/y-/z-axis acceleration, pitch, roll, power, and overall total energy. Regardless of the agreement between the evaluators in identifying an ’eating’ IU and the length of the time window, the acceleration along the x-axis (for both the source of the raw data stratifications) shows the highest rate of median variable importance across the time-widths considered. On the contrary, the power and total energy of the movement seem to be the less important variables in all stratifications (Figure 3).

**Figure 4:**
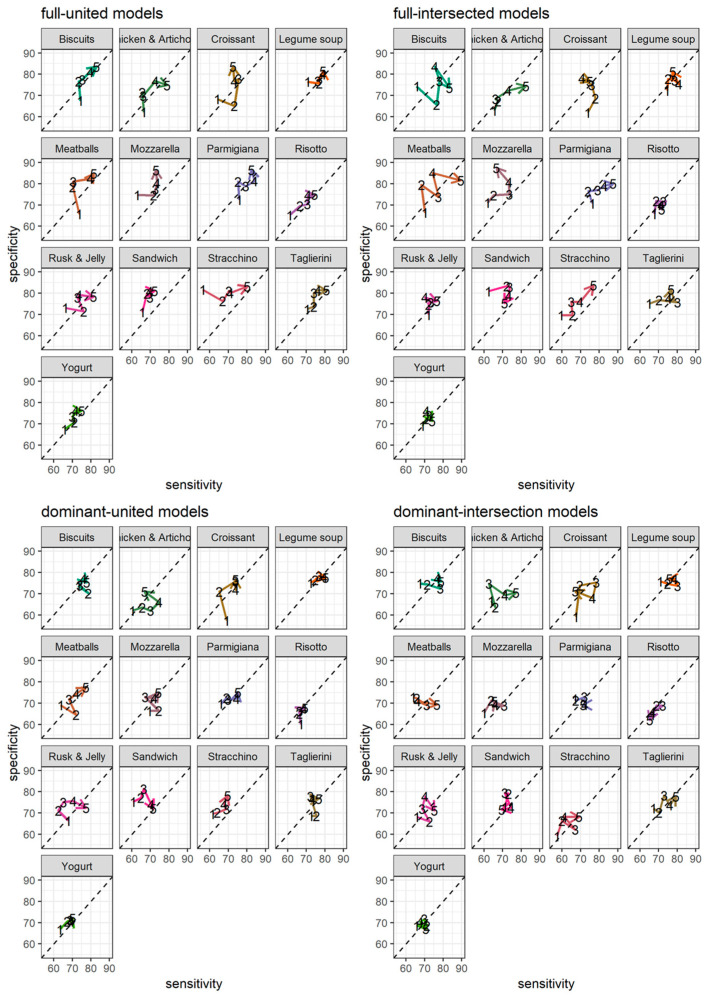
Sensitivity (on x-axes) and specificity (y-axes) for the twenty trained random forest models stratified by the source of raw data (i.e., full or dominant, by panels’ rows), by labeling criteria (i.e., united or intersected, by panels’ columns), by time-width (i.e., the number corresponding to each dot in each sub-panel plot is the time-width in seconds of the window used to train the corresponding model), and by food item (i.e., sub-panel plots). Paths connecting each set of points are oriented from points representing the performances of models stratified on 1 s time-widths to the ones of models stratified on 5 s time-widths.

The average increase in performance by increasing the time-width of the windows is apparent almost everywhere (Figure 4 and Supplementary Tables S1–S4). Thus, there are cases in which extending the time-width from 3 to 5 s improves specificity at the expense of sensitivity (e.g., croissant for both the models stratified with full source of raw data) or vice versa (e.g., rusk and jelly or sandwich for both the models stratified with dominant source of raw data). RF classifiers achieved the highest performance in identifying meatballs (89.4% sensitivity and 81.6% specificity).

The differences in performance between RFs stratified by the full or dominant sources of raw data are not very large. However, improvements, when the stratification was on the full source of raw data, are evident for the classification of food being eaten moving both hands actively (e.g., meatballs, mozzarella, sandwiches, stracchino, and taglierini). Instead, when the stratification considers the dominant source of raw data, the models lead to less variable results among the different time-widths, even if they were generally worse.

The difference in performance between RFs stratified by the different label criteria, i.e., intersected and united, exhibits some patterns only for the stratification on the dominant source of raw data (e.g., in meatballs, the models stratified by united label criteria show increasing sensitivity and specificity as the time-width increases as opposed to the only increase in sensitivity when the stratification was by intersected label criteria). However, stratifying by intersected label criteria generally flattens the growth of the specificity in favor of the sensitivity.

## 4. Discussion

In recent years, significant research has been focused on wearable sensors and devices for monitoring human activities [19,20]. Automatic detection of food intake is essential for understanding people’s eating behavior and overcoming the current limitations of self-reported food intake. Despite advancements over the years, most proposed techniques have been unsuitable for regular use, involving multiple on-body sensors [21].

The first contribution of this paper is that it describes a method to classify food items under semi-natural eating conditions using raw data from the smartwatch-oriented accelerometer sensor. The use of raw sensor data is commonplace in many other biomedical applications, e.g., these are an effective tool for characterizing physical activity patterns in free living [22,23]. Amft and colleagues studied the use of inertial sensors worn on the body to monitor eating and drinking activities as well as the recognition of various eating-related gestures, such as eating from a plate with a fork and knife, eating from a bowl with a spoon, eating with hands, and drinking from a glass [24,25,26]. In a related work, they recognized gestures specific to 11 food categories [27]. Päßler et al. developed a method to recognize food intake activity with different types of foods using the classification of sounds [28]. Y. Dong and colleagues devised a technique to identify and count food bites and beverage sips consumed during a meal [29]. All these methods progress toward using body-worn sensors to automatically identify eating activity, but applying them at the population level is not feasible. Our study setting suggests suitable methods for dietary monitoring at a population level. The study results suggest that monitoring food items using simple wrist-banded wearable devices is feasible but could be more accurate. Using a time-width of 5 s for the windows and dominant source of raw data provides more accurate and stable results for most food items under evaluation for both labeling strategies.

A second contribution of this paper is that this study highlights that the use of machine learning can also be promising for the detection of food items during eating activities, i.e., meals. The RF classifier reaches the highest performance in identifying meatballs (89.4% sensitivity and 81.6% specificity) and the lowest in identifying sandwiches (74.6% sensitivity and 72.5% specificity). Foods such as parmigiana (87.5% sensitivity and 80.0% specificity) and biscuits (83.4% sensitivity and 73.6% specificity) showed relatively stable performance, while artichoke and chicken (82.5% sensitivity and 74.4% specificity) and taglierini (80.8% sensitivity and 81.4% specificity) showed moderate performance.

When compared to previous studies, our results demonstrate significant advancements and competitive accuracy. For example, Amft et al. identified 11 distinct foods consumed by a single individual with 80% accuracy [27], and Y. Dong et al. achieved 86% sensitivity in detecting bites during meals [29]. This represents an improvement compared to previous studies that limit themselves to counting only the number of bites and cannot completely fill the gap between recognition of the bites and their respective energy contribution [4,30,31,32,33]. Similarly, Thomaz et al. achieved F-scores of 76.1% using wrist-worn inertial sensors to detect eating episodes but did not classify individual food items [21]. Bi et al. used acoustic sensors, achieving 84.9% accuracy in identifying food categories, though their method was limited by susceptibility to background noise interference [32]. In contrast, our accelerometer-based approach achieves comparable accuracy while avoiding privacy concerns and noise-related limitations.

More recently, Pouladzadeh et al. employed image-based systems and achieved an average accuracy of 92.21% in identifying specific food items from meal photographs [3]. However, their method relies heavily on user participation to capture high-quality images, which may not always be practical in natural eating environments. In comparison, our wrist-worn accelerometer approach offers a passive, hands-free, and scalable solution, making it more suitable for long-term adoption and population-level dietary monitoring.

Another contribution of this paper is that this study has been considered as the first step in a two-step procedure in which we have tested the real possibility of using raw accelerometer data to classify foods. The next step should be to add a reference kcal value conditional on the recognized food item. We chose to select an integer number of seconds as units for the windows’ time-widths because Fenix^®^ 5 records data at 5 Hz but stores them in memory with a frequency of 1 Hz grouping five values together in a single internal record. Therefore, the entire project is designed to be adaptable and scalable for the development of real-time recognition of caloric intake (kcal) based on Fenix^®^ watches.

We acknowledge that the entire system should improve its performance before the real-time implementation and that future work will require a broader range of food items and a larger sample size.

## 5. Conclusions

This study investigates the feasibility of classifying food items under semi-natural eating conditions using raw accelerometer data from wrist-worn wearable devices. Applying machine learning, specifically random forest classifiers, the method achieved promising results, with high sensitivity and specificity for certain food items such as meatballs. However, the performance varied across food items, highlighting areas for improvement. Compared to earlier approaches, this method is simpler, less invasive, and more suitable for large-scale dietary monitoring.

The results suggest that wearable devices can play a key role in improving how we monitor eating behaviors and calorie intake, offering a practical alternative to self-reported methods. This research is an important first step, and future work will focus on adding calorie estimation, testing with a wider variety of foods, and involving more participants to make the system even more accurate and useful in real-world settings.

## 6. Limitations

The main limitation of this study is the small sample size and we consider only young subjects aged 20–30 years. Another limitation of this study is windowing based on fixed time-widths. This technique offers a simpler approach to learning activity models during training. This is a common approach with raw kinetic data at a constant rate from the sensors [34]. However, one has to deal with the problem of selecting a single, optimal time-width for models. If a very small time-width is chosen, there is a possibility that it will not contain any relevant information for making any useful decision. If the time-width is too wide, the information about multiple activities can be embedded into it, and the activity that dominates the time interval will have a greater influence on the classification decision. Much heuristic information, such as the mean length of the activities and sampling frequency of the sensors, can be employed to determine the optimal time-width. However, the best approach would be to automatically derive the time-width using a data-driven approach. Moreover, different eating movements can span windows of different time-widths. As a part of future work, we will evaluate the application with diverse populations (e.g., older adults and those with different eating habits), exploring dynamic time windows for improved accuracy, and addressing current limitations such as sample size and fixed time-widths.

## Figures and Tables

**Table 1:** Characteristics of study participants: the values represent the mean (standard deviation) of characteristics of participants by gender.

Characteristics	Women (*n* = 9)	Men (*n* = 11)	All (*n* = 20)
Age (year)	23.33 (3.24)	23.45 (3.45)	23.40 (3.27)
Weight (kg)	59.80 (5.22)	76.36 (8.96)	68.91 (11.19)
Height (cm)	166.22 (5.67)	181.36 (6.12)	174.55 (9.64)
BMI (kg/m^2^)	21.67 (1.83)	23.25 (2.83)	22.54 (2.50)
Waist circumference (cm)	69.78 (4.29)	76.91 (5.60)	73.70 (6.12)
Hip circumference (cm)	93.06 (4.19)	94.64 (6.16)	93.92 (5.29)
Bicipital skinfold (mm)	7.64 (3.04)	5.25 (1.80)	6.33 (2.66)
Tricipital skinfold (mm)	18.13 (4.37)	11.95 (4.75)	14.73 (5.47)
Subscapular skinfold (mm)	15.18 (5.41)	14.58 (5.71)	14.85 (5.44)
Suprascapular skinfold (mm)	13.56 (3.82)	11.60 (3.56)	12.48 (3.72)

**Table 2:** Food-items composition. Menus A and B each one made up four meals (i.e., Breakfast, Lunch, Snack, and Dinner) were randomly assigned to the 20 volunteers recruited as participants in this study. The number of original sensor’s records recorded by Fenix^®^ among all subjects and meals stratified by food item are reported under the raw data column.

ID	Food Item	Meal	Energy/Serving (kcal)/(g)	Menu	Raw Data
01	Rusks and Jelly	Breakfast	114/41	A	15,830
02	Yogurt	Breakfast	186/170	A/B	40,114
03	Croissant	Breakfast	99/28	B	9224
04	Risotto	Lunch	471/300	A	32,422
05	Taglierini	Lunch	546/300	B	30,458
06	Mozzarella	Lunch	242/100	A	15,450
07	Meatballs	Lunch	135/120	B	11,240
08	Biscuits	Snack	278/55	A	22,090
09	Sandwich	Snack	195/70	B	17,652
10	Legume soup	Dinner	135/310	A	19,034
11	Artichoke and Chicken	Dinner	199/120	A	17,984
12	Parmigiana	Dinner	309/300	B	31,436
13	Stracchino	Dinner	269/100	B	15,326

**Table 3:** Dataset’s dimensions and characteristics. For each row in the table, the number of windows for the corresponding stratified analysis among all the 278,260 pieces of raw data initially retrieved by the devices are reported under the Information Units (IUs) column. The number of IUs used to train the corresponding random forest classification model, i.e., the number of IUs labeled as ’eating‘, are reported under the ’eating‘ labels column. The number of kinetic variables used for the corresponding analysis are reported under the kinetic variables.

Source	Label Criteria	Time-Width (s)	Information Units	’Eating’ Labels	Kinetics Variables
full	united	1	27,798	8567	14
full	united	2	27,718	8171	14
full	united	3	27,638	8599	14
full	united	4	27,558	7921	14
full	united	5	27,478	7713	14
full	intersected	1	27,798	4776	14
full	intersected	2	27,718	4335	14
full	intersected	3	27,638	4627	14
full	intersected	4	27,558	3122	14
full	intersected	5	27,478	2286	14
dominant	united	1	13,899	8567	7
dominant	united	2	13,859	8171	7
dominant	united	3	13,819	8599	7
dominant	united	4	13,779	7921	7
dominant	united	5	13,739	7713	7
dominant	intersected	1	13,899	4776	7
dominant	intersected	2	13,859	4335	7
dominant	intersected	3	13,819	4627	7
dominant	intersected	4	13,779	3122	7
dominant	intersected	5	13,739	2286	7

## Data Availability

The original contributions presented in the study are included in the article/Supplementary Material, further inquiries can be directed to the corresponding author.

## Supplementary Materials

The following supporting information can be downloaded at: https://www.mdpi.com/article/10.3390/foods14020276/s1, Table S1: Sensitivity/specificity for food classification based on a Random Forest classification model stratified on full source of raw data and intersected labelling criteria; Table S2: Sensitivity/specificity for food classification based on a Random Forest classification model stratified on dominant source of raw data and intersected labelling criteria; Table S3: Sensitivity/specificity for food classification based on a Random Forest classification model stratified on full source of raw data and unioned labelling criteria; Table S4: Sensitivity/specificity for food classification based on a Random Forest classification model stratified on dominant source of raw data and unioned labelling criteria.

## Abbreviations

CSV	Comma Separate Value
CV	Cross-Validation (CV)
FIT	Flexible and Interoperable Data Transfer
FPS	Frame Per Second
IU	Information Unit
NCDs	Noncommunicable Diseases
NOTION	measuriNg calOric inTake at populatION level
RF	Random Forest
