# Supplementary material for: Classifying Food Items During an Eating Occasion: A Machine Learning Approach with Slope Dynamics for Windowed Kinetic Data
Source: Foods. 2025 Jan 16;14(2):276. doi: 10.3390/foods14020276 (PMC11764823; doi:10.3390/foods14020276)
Supplement: Supplementary file 1 [file foods-14-00276-s001.zip › foods-3382837-supplementary.pdf]

Supplementary materials

Table S1. Sensitivity/specificity for food classification based on a Random Forest classification model stratified on full source of raw data and intersected labelling criteria. The value of the mtry represents the optimal number of variables used by each tree of the forest to grow as determined by the 5-fold cross-validation procedure.

**Time-width**	**mtry**	**Rusks and Jelly (%)**	**Yogurt (%)**	**Brioche (%)**	***Risotto* (%)**	***Taglierini* (%)**	***Mozzarella* (%)**	**Meatballs (%)**	**Biscuits (%)**	**Sandwich (%)**	**Legume soup (%)**	**Artichoke Chicken (%)**	***Parmigiana* (%)**	***Stracchino* (%)**
1	42	72.1/69.8	68.9/69.1	73.8/62	67.7/65.8	64.9/75.1	61.8/71	70.2/66.3	65.7/74.3	62.3/80.9	74.5/72.3	65.6/62.9	76.3/70.7	59.5/69.7
2	5	72.3/74.6	70.7/72.3	**78**/68.6	68.7/**71.9**	70.2/76.8	65.2/74.6	68.3/79.4	76.5/65.6	72.2/**83.3**	75.1/77.8	67.6/67.3	74.1/76.5	65.6/69.6
3	52	73.4/75.7	72.3/73.9	76/74.9	**72.5**/71.6	**80.1**/75.7	**73.6**/75	77.5/73.7	78/76.8	**73.8**/82.9	78.6/77.1	66.2/68.4	79.3/77	65/76
4	24	70.2/78	71/76	70.8/**78.1**	71.5/69.9	75.5/77.5	73.4/80.7	74.3/**85.1**	75.6/**83.6**	73.4/78.9	**80.9**/75.3	73.1/72.3	82.7/79	69.8/75.9
5	25	**76.6/76.3**	**74.1/71.3**	75/76.7	71.6/67.9	76.7/**81.6**	66.7/**87.4**	**89.4**/81.6	**83.4**/73.6	71/75.3	77.5/**81.2**	**82.5/74.4**	**87.5/80**	**76.9/83.6**

Table S2. Sensitivity/specificity for food classification based on a Random Forest classification model stratified on dominant source of raw data and intersected labelling criteria. The value of the mtry represents the optimal number of variables used by each tree of the forest to grown as determined by the 5-fold cross-validation procedure.

**Time-width**	**mtry**	**Rusks and Jelly (%)**	**Yogurt (%)**	**Brioche (%)**	***Risotto* (%)**	***Taglierini* (%)**	***Mozzarella* (%)**	**Meatballs (%)**	**Biscuits (%)**	**Sandwich (%)**	**Legume soup (%)**	**Artichoke Chicken (%)**	***Parmigiana* (%)**	***Stracchino* (%)**
1	3	65.8/68.2	65.6/68.6	67.5/59.1	66.8/64.5	67.2/72.2	60/65.4	63.8/**72.9**	67.4/74.8	71.2/72.9	70.8/75.8	63.9/66.6	66.9/72.1	57.4/59.2
2	2	72.6/66	**71.3**/68.5	69.5/73.9	68.9/**68.9**	70.2/70.2	63.8/**71.2**	65.8/72.3	71.4/74.4	73.1/79.1	74.9/74.7	66.2/63.6	67.4/71.2	60/66.3
3	4	68.7/72	70/**71.7**	**78.4/75.3**	**72.3**/68.8	72.9/**78.2**	**70.8**/68.8	71.1/69.1	78.4/72.4	71.2/**79.4**	**80.1**/73.7	62.6/**74.7**	72.1/**73**	67.1/62.2
4	3	70.2/**77.8**	67.6/70.4	76.4/67.9	66.8/65.8	76.1/73.7	66.2/69.4	66.2/71	76.9/**77.2**	**74.6**/72.5	78.6/**77**	71.3/69.3	**72.4**/69.2	61.1/68.1
5	2	**75.3**/71.2	70.7/67	67.1/71.2	65.2/62.1	**79.3**/77.8	65.5/70.9	**76.6**/69.3	**78.8**/75.7	69.5/71.6	75.7/76.9	**77.2**/70.5	71.4/69.2	**69.2/68.2**

Table S3. Sensitivity/specificity for food classification based on a Random Forest classification model stratified on full source of raw data and unioned labelling criteria. The value of the mtry represents the optimal number of variables used by each tree of the forest to grown as determined by the 5-fold cross-validation procedure.

**Time-width**	**mtry**	**Rusks and Jelly (%)**	**Yogurt (%)**	**Brioche (%)**	***Risotto* (%)**	***Taglierini* (%)**	***Mozzarella* (%)**	**Meatballs (%)**	**Biscuits (%)**	**Sandwich (%)**	**Legume soup (%)**	**Artichoke Chicken (%)**	***Parmigiana* (%)**	***Stracchino* (%)**
1	2	66.3/73.1	65.8/67.4	64.1/68.3	61.1/65.2	70.4/72.5	63/74.7	73.7/65.7	74.3/67.5	65.7/71.1	70.6/76.5	66.5/62.2	75.8/72.4	56.2/81.6
2	5	76.1/71.4	71.2/70.8	73.3/65.1	66.6/69.6	74/73.7	71.8/74.5	70.3/78.4	73.5/76.6	68.2/79.8	77/75.7	65.7/71.4	75.2/81	67/76.3
3	2	72.8/77.9	70/73.5	**75.6**/77.8	70.5/70.4	74/80.1	72.7/77.4	70.6/81	75.4/77.5	69.8/78	76.2/76.8	66.1/69.9	79.4/78.8	70.3/81
4	4	73.6/**79.2**	71.7/**76.2**	73.2/76.7	70.9/74.6	**76.2**/81.3	**73.6**/80.3	79.7/82.4	79.6/81.1	69.7/80.8	**79**/79.9	72.7/**76.8**	**84.1**/81.3	71.1/80.1
5	5	**81.4**/78.3	**74.9**/76.1	72.5/**83.6**	**74.3/74.8**	80.8/**81.4**	73/**86.3**	**81.5/84.7**	**82.9/83.5**	**72.2/81**	**79/81.4**	**79**/74.8	82.4/**86.5**	**80.2/83.2**

Table S4. Sensitivity/specificity for food classification based on a Random Forest classification model stratified on dominant source of raw data and unioned labelling criteria. The value of the mtry represents the optimal number of variables used by each tree of the forest to grown as determined by the 5-fold cross-validation procedure.

**Time-width**	**mtry**	**Rusks and Jelly (%)**	**Yogurt (%)**	**Brioche (%)**	***Risotto* (%)**	***Taglierini* (%)**	***Mozzarella* (%)**	**Meatballs (%)**	**Biscuits (%)**	**Sandwich (%)**	**Legume soup (%)**	**Artichoke Chicken (%)**	***Parmigiana* (%)**	***Stracchino* (%)**
1	2	67.8/66.4	63.4/66.8	69.1/57.4	67.1/60.1	72.6/68.6	69.5/66.4	63.7/69.1	73.2/73.7	60.7/75.3	72.3/75.2	60.4/62.1	66/69.8	61.4/69.6
2	2	62.5/71.2	67.3/70.6	65.2/71	66.2/65.1	75.4/68.6	**74.6**/66.5	72.1/64.5	**78.6**/70.2	64.9/77.1	74.8/76.5	65.7/63.5	69.1/72.5	64/70.2
3	2	65.2/75.1	68.8/71.1	**73.9**/75.9	66.2/66.2	72.3/**77.8**	67.2/72.9	68.2/71.8	74/73.9	66.4/**81.4**	76.8/**78.3**	70.2/62	69.8/71.7	69/71.9
4	4	70.5/**75.6**	69.4/70.5	**73.9**/71.9	68.7/67.5	73.3/76.5	72.1/72.3	72.2/74.5	76/**77**	70.4/74	79.6/77.6	**74.7**/66	74.4/72.1	67.7/74.1
5	4	**77.3**/72.4	**70.1/72**	73.8/**76.6**	**69.2/67.7**	**76.6**/76.7	74.3/**74.9**	**77.3/77.4**	77.7/75.3	**71.5**/72.1	**80.4**/77.5	66.9/**71**	**75.1/75**	**69.5/78.1**
